# Effectiveness of enhanced recovery after surgery in elderly hepatolithiasis patients after partial hepatectomy

**DOI:** 10.12669/pjms.41.11.12718

**Published:** 2025-11

**Authors:** You Peng, Huan Wan, Xiahong Hu, Ying Wang, Yi Cao

**Affiliations:** 1 You Peng The Second Department of Biliary Surgery, Hunan Provincial People’s Hospital, The First Affiliated Hospital of Hunan Normal University, Changsha city, Hunan Province 410000, P.R. China; 2 Huan Wan Department of Nursing, Hunan Provincial People’s Hospital, The First Affiliated Hospital of Hunan Normal University, Changsha city, Hunan Province 410000, P.R. China; 3 Xiahong Hu The Second Department of Biliary Surgery, Hunan Provincial People’s Hospital, The First Affiliated Hospital of Hunan Normal University, Changsha city, Hunan Province 410000, P.R. China; 4 Ying Wang, Hunan Provincial People’s Hospital, The First Affiliated Hospital of Hunan Normal University, Changsha city, Hunan Province 410000, P.R. China; 5 Yi Cao The Second Department of Biliary Surgery, Hunan Provincial People’s Hospital, The First Affiliated Hospital of Hunan Normal University, Changsha city, Hunan Province 410000, P.R. China

**Keywords:** Enhanced recovery after surgery, Effectiveness, Elderly, Hepatolithiasis, Partial hepatectomy

## Abstract

**Objective:**

Enhanced Recovery After Surgery (ERAS) aims to reduce patients’ stress response during the perioperative period through a series of evidence-based optimization measures, promoting rapid rehabilitation. This study aimed to evaluate the effectiveness and safety of ERAS in this high-risk group, with particular attention to the recovery of liver function, gastrointestinal function, and postoperative complications, all of which are critical to improving outcomes in elderly patients and have not been thoroughly investigated in previous studies.

**Methodology:**

The clinical data of elderly patients with hepatolithiasis who underwent partial hepatectomy in Hunan Provincial People’s Hospital from October 2022 to October 2024 were retrospectively analyzed. Patients were divided into the ERAS and the control groups according to whether they received ERAS during the perioperative period. The perioperative indexes, liver function indexes, and complications were compared between the two groups.

**Results:**

A total of 141 patients met the inclusion criteria: 73 patients in the ERAS group and 68 patients in the control group. After operation, the first exhaust time and postoperative hospital stay in the ERAS group were significantly lower than in the control group (P<0.05). Five days after the operation, levels of aminotransferase (ALT) in the ERAS group were significantly lower, and albumin (ALB) levels were significantly higher than those in the control group (all P<0.05). ERAS was associated with considerably lower total number of postoperative complications (P<0.05).

**Conclusions:**

The application of the ERAS concept in the perioperative period of partial hepatectomy in elderly patients with hepatolithiasis is safe and effective.

## INTRODUCTION

Hepatolithiasis is a medical condition, predominantly prevalent in Asia, characterized by the development of calcium bilirubinate and cholesterol stones in the intrahepatic bile ducts.^1^ With the gradual aging of the general population, the prevalence of hepatolithiasis in the elderly patients is on the rise.^1^-^3^ Hepatectomy remains one of the important methods of treating hepatolithiasis.^4^ However, due to the decline of physical function and complications, the perioperative nursing of elderly hepatolithiasis patients is challenging.^1^,^5^ The current routine nursing in the perioperative period is associated with some adverse effects, such as a risk of dehydration due to long preoperative fasting time, deep vein thrombosis, pulmonary infection due to the extended postoperative time lying in bed, or slower recovery of patients’ Intestinal function that may impact their nutritional status and rehabilitation.^5^-^7^

Adequate science-based perioperative management is crucial in reducing the postoperative complications of liver surgery.^6^,^7^ The concept of rapid rehabilitation surgery (ERAS) has been widely used to reduce the stress response and complications of surgery, and to accelerate the rehabilitation.^6^-^9^ The ERAS concept optimizes a systematic perioperative intervention, including strengthening preoperative education, canceling preoperative bowel preparation, shortening preoperative fasting time, and not indwelling gastric tube, performing intraoperative thermal insulation and local infiltration anesthesia combined with high epidural and general anesthesia, to avoid fluid overload, sufficient postoperative analgesia, early postoperative ambulation, early postoperative eating, etc.^7^-^10^ Since the concept of ERAS was put forward, its benefits were demonstrated in a variety of surgical procedures, including gynecological, gastrointestinal, and orthopedic surgeries.^8^,^9^

Elderly-specific considerations: Beyond the general benefits of ERAS, elderly patients present distinct physiological challenges that can hinder postoperative recovery. Age-related immunosenescence impairs both innate and adaptive responses, increasing susceptibility to postoperative infections; sarcopenia and malnutrition reduce muscle strength and delay mobilization and functional recovery; moreover, the incidence of perioperative complications—including pulmonary infection, deep vein thrombosis, bile leak, and delayed hepatic functional recovery—tends to be higher in elderly cohorts than in younger patients.^9^-^11^ In elderly patients with hepatolithiasis, chronic biliary inflammation and limited hepatic reserve may further exacerbate these risks, underscoring the need to tailor ERAS elements to this high-risk population.^10^,^11^

In 2008, the ERAS concept was applied to nursing patients undergoing hepatectomy.^10^ Although ERAS has been widely applied in various surgical procedures, its role in elderly patients with hepatolithiasis undergoing partial hepatectomy remains underexplored. This population faces unique physiological challenges, including limited hepatic reserve, immunosenescence, sarcopenia, and an increased risk of postoperative complications. Therefore, this study aimed to evaluate the effectiveness and safety of ERAS in this high-risk group, with particular focus on clinically meaningful short-term outcomes such as gastrointestinal recovery, liver function recovery, and postoperative complications, which are critical to improving outcomes in elderly patients but have not been thoroughly investigated in previous studies.

## METHODOLOGY

The clinical data of 141 elderly patients (over 60 years old) who underwent hepatectomy for hepatolithiasis at Hunan Provincial People’s Hospital from October 2022 to October 2024 were retrospectively analyzed.

### Inclusion criteria:

- Age ≥ 60 years old.
- The diagnosis of hepatolithiasis was confirmed by imaging examination.
- The liver function Child Pugh grade A or B.
- ASA (American Society of Anesthesiologists) I-III physical condition.

### Exclusion criteria:

- Severe hepatic dysfunction: Child–Pugh class C (preoperative assessment).
- Acute cardiovascular/cerebrovascular events: myocardial infarction or stroke within 3 months, or decompensated heart failure (NYHA III–IV) requiring inotropic support.
- Severe renal insufficiency: eGFR < 30 mL/min/1.73 m^2^ or maintenance dialysis.
- Need for ICU-level care postoperatively: patients transferred to the ICU immediately after surgery or requiring invasive mechanical ventilation > 24 h.
- Active uncontrolled sepsis at the time of surgery, or other conditions precluding ERAS implementation as judged by the treating team.
- Concomitant malignant tumors or incomplete key variables for the primary outcomes.

A total of 73 patients who were nursed using the ERAS concept during the perioperative period were selected as the ERAS group, and 68 patients who received routine nursing were selected as the control group.

### Ethical approval:

The research protocol was reviewed and approved by the Medical Ethics Committee of Hunan Provincial People’s Hospital (Approval No: LY-2025-173; Date: May 23, 2025). All procedures performed in this study were in accordance with the ethical standards of the institutional and/or national research committees, as well as with the 2013 Helsinki Declaration and its subsequent amendments. Given the retrospective and observational nature of this study, the medical ethics committee waived the requirement for informed consent.

### Perioperative management methods:

The control group received routine nursing management, while the ERAS group followed the intervention measures of the ERAS concept during the perioperative period. In the ERAS group, early postoperative mobilization and early feeding were emphasized as key measures. Patients were encouraged to sit up and ambulate with assistance on the first postoperative day, starting with 10–15 minutes of bedside standing or walking and gradually progressing to independent ambulation as tolerated. Regarding feeding, within 6–12 hours after surgery, patients were allowed small amounts of glucose water or clear fluids; on the first postoperative day, they were advanced to semi-liquid diets and subsequently transitioned to soft or regular diets according to tolerance. These interventions were intended to accelerate gastrointestinal recovery, improve nutritional status, promote albumin synthesis, reduce insulin resistance, and prevent complications such as pulmonary infection and deep vein thrombosis. In addition, standardized preoperative education and psychological support were provided in the ERAS group. Specifically, trained nursing staff conducted one-to-one counseling sessions 24–72 hours before surgery to explain the surgical process, the ERAS pathway, and the expected recovery timeline. Printed take-home materials were distributed to patients and caregivers, and patients practiced breathing exercises and low-intensity cardiopulmonary training under supervision. Given the retrospective nature of this study, no validated psychological scales were collected; however, these interventions aimed to alleviate preoperative anxiety and fear and to improve adherence to early mobilization and feeding. The specific intervention procedures of the two groups are listed in Table-I.

**Table-I:** ERAS protocol and routine nursing intervention procedure.

Perioperative period	ERAS	Routine nursing
Preoperative		
Patient education	One-to-one preoperative counseling by trained nurses (24–72 h before surgery); provision of printed ERAS timeline and recovery goals to patients/caregivers; supervised breathing exercises and low-intensity cardiopulmonary training; psychological support to alleviate anxiety and enhance adherence. No routine use of validated scales due to the retrospective design.	Conventional education. Patients are not deliberately instructed to exercise cardiopulmonary function.
Fasting, water	Fasting solid food 6 hours before operation and drinking 50ml of 50% high sugar 2 hours before operation.	Fasting and drinking were forbidden 12 hours before the operation.
Bowel preparation	No bowel preparation.	A laxative or a clean enema is used one night before the operation.
Indwelling gastric catheter and urinary catheter	The gastric tube is not retained, and the urinary tube is retained after anesthesia and before operation.	A gastric tube and urinary catheter are indwelled after anesthesia and before the operation.
Intraoperative		
Anesthesia mode	Local infiltration anesthesia combined with high epidural and general anesthesia.	General anesthesia.
Intraoperative heat preservation	An electric blanket and heated infusion set are used; warm water at about 39°;C is used for rinsing.	None.
Restricted infusion	The abdominal cavity is washed.	None.
Postoperative	The infusion volume, especially the input of salt, is controlled	
Analgesia		Intravenous analgesia pump, analgesia, combined with injection of opioid analgesics.
Early feeding	Multimodal analgesia, such as high epidural analgesia combined with NSAID injection.	After the patient is ventilated, the juice will flow.
Early activities	On the first day after the operation, an appropriate amount of glucose water is injected, and gradually transferred to half-flow.	Patients are not asked to get out of bed within 1 day after the operation.
Catheter removal	Ambulation is encouraged on the first day after the operation.	After ventilation, the gastric tube is removed, the urinary catheter is clamped on the first day after operation to exercise bladder function, and the urinary catheter is removed on the second or third day after operation.
Discharge criteria	Patients return to the ward after the operation, the urinary catheter is pulled out after waking up, and the abdominal drainage tube is removed as soon as possible, according to the patient’s condition.	Same as the ERAS concept.

### Outcome indicators:

The outcome indicators of the two groups, including operation duration, blood loss, exhaust time, length of hospital stay (LOS), liver function test indicators, and complications (evaluated by Clavien-Dindo classification), were compared. Liver function indexes included alanine aminotransferase (ALT), aspartate aminotransferase (AST), total bilirubin (TBIL), and albumin (ALB).

### Statistical analysis:

The data were statistically analyzed by SPSS 25.0 software (IBM Corp., Armonk, NY, USA). The normal distribution of continuous variables was evaluated by the Shapiro-Wilk test. If data conformed to the normal distribution, it was expressed as the mean and standard deviation (SD), and a t-test was used. The non-normal distribution was described by the median and interquartile interval (IQR), which was analyzed by the Wilcoxon test. Categorical variables were reported in the form of frequency and percentage, and the chi-square test was used to test the differences between the two groups as appropriate. P<0.05 indicated significance. All reported P values were bilateral. All perioperative and postoperative data were obtained from the hospital’s electronic medical record system. Prior to statistical analysis, two independent researchers performed data cleaning and cross-checking to ensure accuracy, completeness, and consistency. No missing or incorrectly recorded values were identified; therefore, no data imputation (e.g., linear or multiple imputation) or sensitivity analyses were necessary.

## RESULTS

A total of 141 elderly patients undergoing hepatectomy were included in this study. Among them, there were 87 males and 54 females, aged 60-86 years, with a median of 68 (65-72) years. There was no significant difference in gender, age, body mass index (BMI), basic diseases, Child Pugh classification, lesion location, and ASA classification between the two groups (P>0.05) (Table-II), indicating good baseline comparability and strengthening the reliability of subsequent analyses.

**Table-II:** Comparison of baseline characteristics between the two groups.

Variables	ERAS group(n=73)	Control group (n=68)	χ^2^/Z/t	P
Male (yes)	44(60.3)	43(63.2)	0.131	0.718
Age (years)	69(65-74)	68(64-71)	-1.626	0.104
BMI	23.2±3.1	24.0±3.2	-1.397	0.165
Hypertension	24(32.9)	25(36.8)	0.235	0.628
Diabetes	14(19.2)	17(25.0)	0.696	0.404
Child Pugh classification of liver function			0.932	0.334
Class A	67(91.8)	59(86.8)		
Class B	6(8.2)	9(13.2)		
Lesion site			1.482	0.477
Choledocholithiasis with left hepatolithiasis	35(47.9)	29(42.6)		
Choledocholithiasis with right hepatolithiasis	15(20.6)	20(29.5)		
Choledocholithiasis with left and right hepatolithiasis	23(31.5)	19(27.9)		
ASA grade			1.696	0428
Grade I	0(0)	1(1.5)		
Grade II	56(76.7)	55(80.9)		
Grade III	17(23.3)	12(17.6)		

The postoperative exhaust time and hospital stay in the ERAS group [25 (20–29) days and 8 (7–8) days, respectively] were significantly shorter than those in the control group [26 (21.5–36.5) days and 8 (7–9), respectively] (both P<0.05) (Table-III). Earlier recovery of bowel function and slightly reduced length of stay are clinically meaningful for elderly patients, as they facilitate earlier ambulation and discharge planning and may reduce downstream complications and resource use.

**Table-III:** Comparison of perioperative conditions between the two groups, M(IQR).

Variables	ERAS group(n=73)	Control group (n=68)	Z	P
Operation time (minutes)	147 (105-165)	154 (104.5-184)	-0.972	0.331
Intraoperative blood loss (ml)	103 (85-190)	104 (81-140)	-0.070	0.944
Exhaust time (days)	25 (20-29)	26 (21.5-36.5)	-2.175	0.030
Postoperative hospital stay (days)	8 (7-8)	8 (7-9)	-2.572	0.010

There was no significant difference in the levels of ALT, AST, TBIL, and ALB between the two groups before the operation, one day, and three days after the operation. Similarly, the levels of AST and TBIL at five days after operation were comparable in the two groups (all P>0.05). On the 5th day after operation, ALT level in the ERAS group [47 (28.7–77.8) U/L] was significantly lower than that in the control group [56.6 (32.1–101.6) U/L], while ALB level was higher [40.1±4.7 vs. 38.2±5.2 g/L] (both P<0.05) (Table-IV). These changes suggest less hepatocellular injury and faster recovery of synthetic function under ERAS, which are especially important in elderly patients with limited hepatic reserve. The total number of reported complications in the ERAS group was significantly lower than in the control group [8/73 (10.96%) vs. 16/68 (23.53%); χ^2^=3.939, P=0.047] (Table-V). This corresponds to an absolute risk reduction of about 12.5% and an approximate number-needed-to-treat (NNT) of eight, indicating that ERAS offers a clinically meaningful improvement in safety and tolerance for elderly patients.

**Table-IV:** Comparison of liver function indexes between the two groups.

Variables	ERAS group(n=73)	Control group (n=68)	Z/t	P
***ALT(U/L)***				
Preoperative	19.65 (9.65-36.17)	16.02 (8.59-35.15)	-1.001	0.317
1-day after surgery	138.1 (119.4-231.3)	134.3 (106.45-203.45)	-0.774	0.439
3-days surgery	101 (79.8-178.7)	105.6 (78.55-143.25)	-0.528	0.597
5-days after surgery	47 (28.7-77.8)	56.6 (32.1-101.6)	-1.983	0.047
***AST(U/L)***				
Preoperative	22.36 (13.12-36.87)	21.43 (13.22-35.77)	-0.411	0.681
1-day after surgery	112.1 (85-156.4)	107.8 (79.5-144.65)	-0.365	0.715
3-days surgery	52.6 (36.4-92.3)	65.2 (45.55-95.8)	-1.343	0.179
5-days after surgery	32.96 (23.54-52.33)	30.08 (22.33-45.69)	-0.811	0.417
***TBIL(umol/L)***				
Preoperative	14.13 (10.84-16.89)	15.73 (9.68-19.05)	-0.980	0.327
1-day after surgery	18.8±7.3	17.8±8.4	0.761	0.448
3-days surgery	19.7±7.4	18.4±8.6	0.917	0.360
5-days after surgery	17.6±6.9	17.9±8.1	-0.291	0.771
***ALB(g/L)***				
Preoperative	40.5±5.6	41.9±6.8	-1.328	0.186
1-day after surgery	37.1±5.4	38.5±6.8	-1.324	0.188
3-days surgery	37.0±4.8	37.7±5.7	-0.840	0.403
5-days after surgery	40.1±4.7	38.2±5.2	2.179	0.031

**Table-V:** Comparison of complications between the two groups, n(%).

Complications	ERAS group(n=73)	Control group (n=68)	χ^2^	P
Total number of complications	8 (10.96)	16 (23.53)	3.939	0.047
Incision infection	2 (2.74)	5 (7.35)	-	-
Bile leakage	1 (1.37)	1 (1.47)	-	-
Subphrenic abscess	1 (1.37)	2 (2.94)	-	-
Pulmonary infection	1 (1.37)	2 (2.94)	-	-
Dislocation of cricoarytenoid joint	1 (1.37)	1 (1.47)	-	-
Postoperative abdominal distension	1 (1.37)	2 (2.94)	-	-
Two or more	1 (1.37)	3 (4.41)		

## DISCUSSION

This study showed that the perioperative ERAS approach in elderly patients with hepatolithiasis undergoing hepatectomy is associated with shorter postoperative exhaust and hospital stay, and fewer complications compared to the routine nursing. These results confirm that ERAS can accelerate intestinal recovery in elderly patients undergoing hepatectomy and has a higher safety profile. Currently, the ERAS concept is widely utilized in the perioperative management of surgery, including hepatectomy, with confirmed safety. A retrospective analysis by Wu et al.^11^ demonstrated that ERAS can effectively reduce the perioperative stress response in patients with liver stones, shorten postoperative hospital stays, and decrease the overall incidence of postoperative complications. Similar results were reported by a study by Zhang et al.^12^ which included a retrospective cohort of 1143 patients and demonstrated that ERAS was safe for patients undergoing hepatectomy, improving their recovery and survival. This study also supports the above conclusions. The incidence of postoperative complications and the mortality rate of elderly patients is high.^13^ The ERAS concept is an evidence-based medical approach that can reduce the stress response caused by surgical trauma and accelerate the rehabilitation of elderly patients.^14^

In this study, there was no significant difference in operation time and intraoperative blood loss between the ERAS approach and the routine nursing intervention, suggesting that ERAS does not increase the risk of hepatectomy. However, the exhaust time and postoperative hospital stay in the ERAS group were shorter than those in the control group. In the ERAS group, the preoperative fasting period was shortened and routine bowel preparation was omitted. Mechanistically, shortened fasting with preoperative carbohydrate loading attenuates postoperative insulin resistance, stabilizes perioperative glucose metabolism, and blunts the surgical stress response, while also reducing patient discomfort (hunger/thirst) and supporting hemodynamic stability.^14^ Omission of bowel preparation prevents unnecessary dehydration and electrolyte derangements—issues particularly relevant in elderly patients—which can otherwise delay early mobilization and impair intestinal recovery.^15^ From a clinical standpoint, preserving fluid–electrolyte balance and mitigating insulin resistance plausibly contribute to faster gastrointestinal function recovery, better early nutritional status (e.g., albumin maintenance), and a lower risk of complications such as postoperative ileus. These physiological effects provide a coherent mechanistic explanation for the earlier return of bowel function, the modest reduction in length of stay, and the lower overall complication rate observed in the ERAS cohort.^16^ In patients who receive ERAS nursing, drinking 50ml of 50% high sugar two hours before operation can eliminate thirst and hunger, reduce postoperative insulin resistance, and reduce anesthesia complications.^13^-^16^ In addition, early postoperative feeding that is adopted in the ERAS approach can promote intestinal peristalsis, shorten anal exhaust time, help to restore intestinal function as soon as possible, and thus shorten the length of hospital stay.^11^,^12^,^15^

Previous studies have demonstrated that ERAS can help mitigate the inflammatory response following liver injury and facilitate liver function recovery.^11^,^17^ This is consistent with the results of this study, which showed lower ALT levels and higher ALB levels in the ERAS group five days after operation. It is plausible that the preoperative education of patients in the ERAS group, aimed at reducing anxiety and fear, and cardiopulmonary exercises to enhance heart and lung function, as well as reduce physical stress response, all contributed to improved recovery and a lower inflammatory response.^11^ Additionally, previous studies demonstrated that early feeding measures can reduce the amount of postoperative infusion, which is conducive to the recovery of gastrointestinal function, nutritional intake, and ALB synthesis.^12^,^17^

The total number of complications in the ERAS group was significantly lower than that in the control group. The occurrence of postoperative complications is related to a variety of factors, including age, underlying diseases, and the skill of the surgeons.^18^,^19^ Although there was no significant difference in the operation itself between the two groups, early postoperative ambulation may promote blood circulation, which is conducive to wound healing and bile excretion.^20^,^21^ Moreover, early ambulation and respiratory exercise promote sputum discharge and reduce the incidence of pulmonary congestion and atelectasis.^18^,^20^ Early eating improves the nutritional status and immunity of patients, and also helps to resist infection.^21^,^22^ This study further confirms the superiority of ERAS compared to the traditional nursing approach in terms of safety profiles, making it a practical choice for elderly patients undergoing hepatectomy.

Beyond physiological optimization, preoperative education in our ERAS pathway was designed to address psychological distress in elderly patients—a group prone to heightened anxiety and reduced treatment adherence. The standardized one-to-one counseling, written take-home information, and supervised breathing/cardiopulmonary exercises were intended to reduce anxiety and fear, thereby improving cooperation with early mobilization and early feeding. This likely contributed to the observed acceleration in gastrointestinal recovery and the lower complication rate. Because of the retrospective design, we did not collect validated psychological scales (e.g., HADS, SAS/SDS), which precluded a quantitative analysis of psychological outcomes; future prospective studies incorporating such instruments are warranted to delineate the mediating role of psychological factors in ERAS benefits.

This study provides additional evidence regarding the application of ERAS in elderly patients with hepatolithiasis undergoing partial hepatectomy, a group that is often at increased perioperative risk and not extensively represented in previous research. The results suggest that ERAS may be safe and clinically beneficial in this population, as reflected by earlier recovery of bowel function, a favorable distribution of hospital stay, better liver function indices by postoperative day-5, and fewer complications. These findings are of practical relevance, as elderly patients frequently have limited hepatic reserve, age-related immune decline, sarcopenia, and higher baseline risk of complications; even modest improvements in recovery may contribute to better functional outcomes and earlier discharge readiness.^18^,^19^,^22^

*Strengths of the present study:* The work include the focus on a well-defined patient group (≥60 years with hepatolithiasis undergoing partial hepatectomy), the use of clear inclusion and exclusion criteria to reduce potential confounding from severe comorbidities, and the assessment of multiple clinically meaningful outcomes. Future studies with larger, multicenter prospective designs, standardized ERAS element reporting, and longer follow-up will be valuable to validate these findings, assess long-term functional recovery and quality of life, and explore underlying mechanisms and cost-effectiveness.

### Limitations

First, it was a single-center retrospective cohort, which introduces the possibility of selection bias and limits causal inference. Second, the sample size was modest, potentially reducing statistical power and restricting the generalizability of the findings. Third, the analysis primarily focused on short-term postoperative outcomes; long-term functional recovery, quality of life, recurrence, readmission, and mortality rates were not assessed. Future studies should include predefined follow-up at 12 and 36 months, using validated instruments such as SF-36 and EQ-5D, along with functional metrics (e.g., activities of daily living), to externally validate and extend our findings. In addition, adherence to individual ERAS elements was not quantified, which may influence outcomes and should be systematically measured in future work. Finally, validated psychological metrics (e.g., HADS, SAS/SDS) were not collected, limiting our ability to quantify the impact of preoperative psychological interventions. Prospective, multicenter randomized controlled trials with larger samples, standardized ERAS-element reporting, and comprehensive long-term follow-up are warranted to confirm the durability, reproducibility, and clinical relevance of ERAS benefits in elderly patients with hepatolithiasis.

## CONCLUSION

The results of this study showed that the application of the ERAS concept in the perioperative nursing of elderly patients with hepatolithiasis undergoing hepatectomy can significantly shorten postoperative exhaust time and the length of hospitalization, improve liver function indicators, and reduce the incidence of complications. Further multicenter studies with larger sample sizes and extended follow-up times are needed to more comprehensively verify the efficacy and safety of the ERAS protocol in this patient group.

### Authors’ contributions:

**YP:** Literature search, study design, manuscript writing, revision, validation and is responsible for the integrity of the study.

**HW, XH, YW and YC:** Data collection, data analysis and interpretation. Critical Review.

All authors have read and approved the final manuscript.
